# Evaluation of Diet Quality, Physical Health, and Mental Health Baseline Data from a Wellness Intervention for Individuals Living in Transitional Housing

**DOI:** 10.3390/nu17152563

**Published:** 2025-08-06

**Authors:** Callie Millward, Kyle Lyman, Soonwye Lucero, James D. LeCheminant, Cindy Jenkins, Kristi Strongo, Gregory Snow, Heidi LeBlanc, Lea Palmer, Rickelle Richards

**Affiliations:** 1 Department of Nutritional Sciences, The Pennsylvania State University, University Park, PA 16802, USA; cjm8279@psu.edu; 2 Department of Nutrition, Dietetics & Food Science, Brigham Young University, Provo, UT 84602, USA; lyman444@student.byu.edu (K.L.); soonwyel@gmail.com (S.L.); james_lecheminant@byu.edu (J.D.L.); 3 Extension Home and Community Department, Utah State University Extension, Utah State University, Logan, UT 84322, USA; cindy.jenkins@usu.edu (C.J.); heidi.leblanc@usu.edu (H.L.); 4 Department of Nutrition, Dietetics & Food Sciences, Utah State University, Logan, UT 84322, USA; kristi.strongo@usu.edu (K.S.); lea.palmer@usu.edu (L.P.); 5 Department of Statistics, Brigham Young University, Provo, UT 84602, USA; snow@stat.byu.edu

**Keywords:** homelessness, transitional housing, SNAP-Ed, nutrition education, diet quality, physical activity, health, wellness intervention, food security, body composition

## Abstract

**Background/Objectives**: The aim of this study was to evaluate baseline health measurements among transitional housing residents (*n* = 29) participating in an 8-week pilot wellness intervention. **Methods**: Researchers measured anthropometrics, body composition, muscular strength, cardiovascular indicators, physical activity, diet quality, and health-related perceptions. Researchers analyzed data using descriptive statistics and conventional content analysis. **Results**: Most participants were male, White, and food insecure. Mean BMI (31.8 ± 8.6 kg/m^2^), waist-to-hip ratio (1.0 ± 0.1 males, 0.9 ± 0.1 females), body fat percentage (25.8 ± 6.1% males, 40.5 ± 9.4% females), blood pressure (131.8 ± 17.9/85.2 ± 13.3 mmHg), and daily step counts exceeded recommended levels. Absolute grip strength (77.1 ± 19.4 kg males, 53.0 ± 15.7 kg females) and perceived general health were below reference standards. The Healthy Eating Index-2020 score (39.7/100) indicated low diet quality. Common barriers to healthy eating were financial constraints (29.6%) and limited cooking/storage facilities (29.6%), as well as to exercise, physical impediments (14.8%). **Conclusions**: Residents living in transitional housing have less favorable body composition, diet, and grip strength measures, putting them at risk for negative health outcomes. Wellness interventions aimed at promoting improved health-related outcomes while addressing common barriers to proper diet and exercise among transitional housing residents are warranted.

## 1. Introduction

Homelessness in the United States (U.S.) has been associated with less healthy diets, including lower than recommended intakes of fiber, calcium, vitamins A and C, fruits, and vegetables [1,2,3,4,5]. Limitations in physical functioning have also been observed among individuals experiencing homelessness and have contributed to low physical activity levels [6]. Negative health outcomes related to poor dietary intake and physical activity shown in this population have included cardiovascular disease and obesity [7,8,9].

Although past studies have attempted to provide a comprehensive health risk assessment in this population, the measurements used in these studies have shown limitations. For example, many studies evaluating dietary intake have focused on nutrient and food group intake rather than overall diet quality [1,2,3,4,5,10]. Diet quality considers an individual’s food intake in comparison with dietary recommendations, which offers a more complete view into a population’s nutritional status [11]. Although Hoisington et al. [12] evaluated diet quality among those experiencing homelessness, the study population focused on U.S. veterans experiencing homelessness, whose experiences may differ from the general adult population experiencing homelessness.

To evaluate body composition in this population, researchers have relied on Body Mass Index (BMI) [2,4,8,9]. Few studies have paired BMI with more specific measures of body composition, such as waist circumference or waist-to-hip ratios [4], which have been shown to be better indicators of health risk than BMI alone [13,14]. Muscular strength has been previously assessed among adults experiencing homelessness in Ireland [6] and Mexico [15] using handgrip strength, which has been shown as an indicator of general health [16]. However, this measure remains unexplored in the U.S.

Limitations from previous studies measuring physical activity levels have included relying on qualitative reports of physical activity or only measuring VO2 max [6,17]. Although one study in the U.S. utilized accelerometers, the purpose was related to evaluating the effectiveness of an intervention aimed at increasing physical activity, not to evaluate normal day-to-day living [18]. Collectively, these methods have prevented researchers from gaining insights about day-to-day physical activity patterns within this population.

Researchers from the present study sought to address the limitations identified in the previous studies by incorporating a more comprehensive set of quantitative and qualitative measures into a pilot wellness (defined by the authors as healthy eating and physical activity patterns aimed at promoting health and well-being) intervention study among adults living in transitional housing units at a local homeless resource center. The aim of the present study was to evaluate baseline measurements from the pilot intervention study including diet quality, body composition (fat mass, fat-free mass, body fat percentage), handgrip strength, accelerometer-based physical activity, blood pressure, heart rate, blood oxygenation levels, health-related quality of life, depression, anxiety, stress, and perceived barriers to healthy living. The breadth of measurements taken, along with the use of validated equipment and measurement approaches, will provide a more complete and accurate view of transitional housing residents’ health status than has been published previously in a single study. Researchers and practitioners can use this comprehensive set of data to more effectively tailor interventions to address health concerns identified. In the present pilot intervention study, we hypothesized that transitional housing residents would have less favorable nutrition and health-related measurements.

## 2. Materials and Methods

### 2.1. Study Design and Population

Researchers utilized cross-sectional data collected from transitional housing residents who took part in an 8-week wellness pilot intervention study at a local homeless resource center. Researchers implemented an adapted Supplemental Nutrition Assistance Program Education (SNAP-Ed) curriculum [19] throughout the 8-week period, with measurements taken before the intervention (week 1), immediately post-intervention (week 10), and two months post-intervention (week 18). The present study reports baseline data collected from four cohorts by researchers between September 2022 and January 2024.

Eligibility criteria included being 18+ years old, English-speaking, and residing in the transitional housing units. Recruitment occurred through posting fliers and in person at a weekly house meeting. Written informed consent was obtained from all participants at baseline, before measurements were taken. Participants received $10 cash for completing the baseline assessment. Utah State University’s Institutional Review Board approved this study (Protocol #12210, approved 5 May 2022, through 15 January 2029).

### 2.2. Data Collection and Measures

#### 2.2.1. Training Protocol

Researchers received training on civil rights, privacy rights, trauma-informed methods [20], and data collection techniques. Data collection training emphasized participant autonomy, offering options for survey completion in private and/or verbal formats and the ability to schedule appointments convenient to participants [20]. Body measurement training emphasized sensitivity, detailing procedures, seeking permission, and refraining from commentary on measurements [20].

#### 2.2.2. Demographics

Participants provided information about age, sex, race, and ethnicity.

#### 2.2.3. Food Security

Participants completed the U.S. Department of Agriculture’s (USDA) Six-Item Short Form of the Food Security Survey Module [21]. Researchers used the USDA scoring criteria to classify food security [21].

#### 2.2.4. Body Temperature, Heart Rate, and Blood Oxygenation

Body temperature was measured with a Therma 9 Pro (Oxiline, LLC., Miami, FL, USA). A Pulse 7 Pro (Oxiline, LLC., Miami, FL, USA) fingertip pulse oximeter was placed on participants’ index finger for 10–15 s to determine heart rate (bpm) and blood oxygen levels (%). Normal levels were considered 97–99 °F for body temperature [22], 60–100 bpm for resting heart rate [23], and 95–100% for blood oxygenation [24].

#### 2.2.5. Blood Pressure

Participants sat with both feet on the ground for five minutes before the measurement [25]. The aneroid sphygmomanometer (American Diagnostic Corporation, Hauppauge, NY, USA) cuff was placed around the upper left arm, and the stethoscope over the brachial artery under the cuff. The cuff was inflated to a maximum of 180 mmHg and released gradually at 2–3 mmHg per second. The first clear sound (Phase 1 Korotkoff sound) indicated systolic blood pressure, while the final distinct sound before muffling (Phase IV Korotkoff) indicated diastolic blood pressure [25]. Blood pressure categories were based on recommendations from the American Heart Association [26].

#### 2.2.6. Anthropometrics

Height was measured using a portable stadiometer (Seca, Hamburg, Germany) without shoes to the nearest 0.1 cm. Weight was measured in normal clothing, without shoes, using a portable digital scale (Befour Inc., Saukville, WI, USA) accurate to ±0.1 kg. BMI was calculated (kg/m^2^), with classifications designated as underweight (<18.5), healthy weight (18.5–24.9), overweight (25.0–29.9), and obese (≥30.0) [27]. Waist and hip circumference were measured at the umbilicus and widest portion of the buttocks, respectively, using a spring-loaded Gulick measuring tape (Blue Jay). Two measurements for each parameter were taken per site, with the average reported. Waist-to-hip ratios (waist circumference/hip circumference) were compared with recommended ranges: <0.9 for males and <0.8 for females [28].

#### 2.2.7. Body Composition

Bioelectrical impedance analysis (Tanita, Tokyo, Japan) provided estimated fat mass (lbs), fat-free mass (lbs), and body fat percentage. Body fat mass index (BFMI, fat mass [kg]/height [m]^2^) and fat-free mass index (FFMI, fat-free mass [kg]/height [m]^2^) calculations and interpretation were based on Kyle et al. [29]. Body fat percentage was compared with sex- and age-specific recommended ranges as outlined by Kyle et al. [29].

#### 2.2.8. Muscular Strength

A Jamar Hydraulic Hand Dynamometer (Patterson Medical, Warrenville, IL, USA) measured handgrip strength, which is an index of overall health [30] and physical function [31]. Participants squeezed the dynamometer at maximal effort with their elbow at a right angle [31]. The highest measure from both hands was summed for absolute grip strength (kg) and then divided by BMI to calculate relative grip strength (kg/BMI) [31]. Values were compared with those reported by Lawman et al. [31] for adult males (absolute grip strength, 89.7 ± 0.8 kg and relative grip strength, 3.2 ± 0.1 kg/BMI) and females (absolute grip strength, 56.1 ± 0.5 kg and relative grip strength, 2.0 ± 0 kg/BMI).

#### 2.2.9. Physical Activity

To determine physical activity, tri-axial Actigraph accelerometers (wGT3X-BT+) (Pensacola, FL, USA) were used. Accelerometers are commonly reported in research studies [32]. Each participant wore the accelerometer on the non-dominant wrist for a period of 7 days. Accelerometers allow for the intensity of activity to be determined. However, given the variability in wrist-worn accelerometer intensity cutpoints reported in previous studies and the lack of validated intensity cutpoints for the population used in the present study, we report only average steps per day. Limitations of wrist-worn intensity cutpoints are discussed in detail by Gao et al. [33]. Step counts were compared to levels shown to reduce all-cause mortality in adults (6000–10,000) [34].

#### 2.2.10. Diet Quality

Diet quality was assessed using the National Cancer Institute’s Automated Self-Administered Dietary Assessment Tool (ASA24) [35] and the Healthy Eating Index-2020 (HEI-2020) [36]. The HEI-2020 measured diet quality based on alignment to the Dietary Guidelines for Americans and yielded a total score between 0 and 100, with higher scores indicating better diet quality [11,36]. The average score among the general US adult population ranges from 57 to 61 [37].

#### 2.2.11. Health-Related Quality of Life (HRQOL SF-36)

Participants’ perceptions of health were based on responses to the eight domains of the HRQOL SF-36 [38]. Scores for each domain were compared to guidelines recommended by the RAND Corporation [38]: Physical functioning, 70.6 ± 27.4; role limitations due to physical health, 53.0 ± 40.8; role limitations due to emotional problems, 65.8 ± 40.7; energy/fatigue, 52.2 ± 22.4; emotional well-being, 70.4 ± 22.0; social functioning, 78.8 ± 25.4; pain, 70.8 ± 25.5; and general health, 57.0 ± 21.1.

#### 2.2.12. DASS-42

The Depression and Anxiety Stress Scale (DASS)-42 included 14 items in each subscale for depression, anxiety, and stress [39]. Response options were 0 = did not apply to me at all, 1 = applied to me to some degree, or some of the time, 2 = applied to me to a considerable degree, or a good part of time, and 3 = applied to me very much, or most of the time. Scores were totaled by subscale, with summed scores classified into normal (depression, 0–9; anxiety 0–7; stress, 0–14), mild (depression, 10–13; anxiety, 8–9; stress, 15–18), moderate (depression, 14–20; anxiety, 10–14; stress, 19–25), severe (depression, 21–27; anxiety, 15–19; stress, 26–33), and extremely severe (depression, 28+; anxiety, 20+; stress, 34+) [39].

#### 2.2.13. Create Better Health Survey

Participants completed an online Qualtrics (Provo, UT, USA) survey using a researcher’s iPad that included questions about stretching food budgets, food variety, nutrition label usage, grocery list usage, food safety adherence, and meal adjustments. Likert-scale response options were 1 (never) to 5 (always). Perceived barriers to healthy eating and physical activity were measured via open-ended questions.

### 2.3. Data Analysis

Researchers used descriptive statistics for Likert-scaled survey items, physical measurements, HRQOL SF-36, DASS-42, and demographics using IBM SPSS, v. 28, and for HEI-2020 and physical activity using R (R Core Team, 2023). Programming from the National Cancer Institute was used to calculate HEI-2020 scores [40]. ActiLife software v6.13.6 (Actigraph, Pensacola, FL, USA) was utilized to initialize, download, and process physical activity data. Physical activity data were originally collected in 10 s epochs but converted to 60 s epochs in the ActiLife software. We excluded any days that did not reach at least 18 h of wear time (75%) [41], as well as participants who did not have at least 2 days of usable data. Using the Actilife software, step counts were determined. Subject-weighted mean scores were calculated. We noted that one participant had an unusually high step count (78,961 steps per day). We note in the Results the average step counts and standard deviations with and without this participant.

For the open-ended questions, researchers used a conventional content analysis adapted from Hseih and Shannon [42]. Two researchers independently reviewed survey responses like a narrative to build a codebook. Researchers independently coded the data on Microsoft Excel (Microsoft 365 subscription) using the codebook. Researchers compared coding and reconciled any discrepancies, with a third researcher resolving any unresolved differences. Total scores and percentages were calculated to identify the most prevalent barriers.

## 3. Results

Most participants identified as male, between 55 and 64 years old, and White, and were classified as having low or very low food security (Table 1).

As shown in Table 2, the mean baseline measurement for blood pressure was 132 ± 17.9/85 ± 13.3 mmHg; BMI was 31.8 ± 8.6 kg/m^2^; waist-to-hip ratio was 1.0 ± 0.1 for males and 0.9 ± 0.1 for females; and body fat mass index was 7.7 ± 3.1 for males and 15.3 ± 7.7 for females. The mean absolute handgrip strength was 77.1 ± 19.4 kg for males and 53.0 ± 15.7 for females. The average number of steps per day taken by participants was 14,334.0 ± 14,297.8 steps for all participants and 11,524.3 ± 3951.6 steps when a single outlier was removed.

Table 3 displays the HEI-2020 data. For the adequacy components, out of 5 maximum points, mean scores were 1.4 for total fruit, 1.3 for whole fruit, 2.4 for total vegetables, 1.9 for greens and beans, 4.2 for protein foods, and 1.5 for seafood and plant proteins. Out of 10 maximum points, the mean scores were 0.8 for whole grains, 6.3 for dairy, and 1.2 for fatty acids. For moderation components, out of a maximum of 10 points, the mean scores were 6.2 for refined grains, 3.9 for sodium, and 7.2 for added sugars. The mean total HEI-2020 score was 39.7 (out of a maximum 100 points).

The mean score for participants’ general health was 52.9 ± 29.8 (Table 4). Mean scores for depression, anxiety, and stress were 8.6 ± 8.7, 9.3 ± 9.3, and 10.9 ± 9.5, respectively. Participants scored lowest on variety (2.3 ± 1.5) and following food safety recommendations (2.5 ± 1.5) and highest on adjusting meals to use foods already available at home (3.7 ± 1.1) and stretching food dollars (3.1 ± 1.5, Table 4). Per the qualitative data, the most common barriers to healthy eating were financial constraints (29.6%) and limited cooking/storage facilities (29.6%), as well as to exercise, physical impediments (14.8%).

## 4. Discussion

The purpose of the present study was to comprehensively evaluate baseline health measures among people living in transitional housing units who participated in a pilot wellness intervention at a local homeless resource center. Resting heart rate, blood oxygenation levels, and body temperature were all in normal or recommended ranges [22,24]. Mean baseline measurements were higher than recommendations for blood pressure, BMI, waist-to-hip ratio, and body fat percentage, whereas handgrip strength and overall diet quality were lower than the reference standards [11,26,27,28,31,36,43]. Average steps per day were higher than other US adults [34]. Qualitative data indicated that the main barriers to healthy eating were finances and limited cooking/storage facilities, and for exercise were physical impediments. Collectively, these results indicated less favorable health risk factors among those transitioning out of homelessness.

The assessment of body composition in the present study indicated less favorable measures, based on recommendations [27,28], with participants having a high average waist-to-hip ratio, indicating central obesity, and an obese BMI. Due to the nature of food insecurity in individuals living in a homeless situation, it has commonly been believed that this population would struggle with being underweight [8]. This is not in fact the case, as 57% of chronically homeless adults in a Tsai and Rosenheck [8] were overweight or obese, with women having the highest risk for obesity. Other studies among adults experiencing homelessness or residing in transitional housing units have similarly shown a high frequency of obesity [2,4,9]. The results from the present study further supported these previous findings, including a higher proportion of females classified as obese than males [9]. However, this study provided unique insights on chronic disease risk through assessing waist-to-hip ratio, fat mass and fat-free mass, and handgrip strength, which were all outside recommended healthy ranges and/or less favorable compared to research samples among US adults [28,29,31]. Further, although disease prevalence was not measured in the present study, participants had higher-than-normal blood pressure (132/85 mmHg) [26]. Previous research has shown a high prevalence of hypertension in people experiencing homelessness [7]; thus, the elevated blood pressure among participants in this study was not surprising. In the present study, the combination of elevated blood pressure with the adverse body composition measures suggests an even higher potential risk for poor chronic disease health outcomes among this population [14,44,45].

Lower mean absolute and relative grip strength compared to the general US adult population was found in the present study, suggesting poorer muscle strength and thus higher risk for frailty syndrome, which is typically associated with aging and risk for adverse health outcomes [31,46]. Prior to the present study, handgrip strength has not been studied among U.S. adults experiencing homelessness. However, two studies have been published on handgrip strength in this population outside of the U.S. [6,15]. In the study done in Ireland, the mean grip strength of people experiencing homelessness was found to be much lower than the general population, even though they were of a younger age [6]. In Mexico, it was found that the measured dominant handgrip strength (34.8 kg) was at a level that indicated frailty syndrome, a condition typically associated with aging adults and people with chronic illnesses [15]. Similar dominant handgrip strength was noted in the present study, suggesting further evidence of potential risk for adverse health outcomes in this population.

Qualitative data showed that the biggest barriers to physical activity for participants were physical impediments. It is possible that participants in this study were thinking of more formal methods of physical activity, such as exercising at a gym, rather than walking, when answering this question. Participants were aware of being given free local recreation center membership as part of participating in the study, so this may have also influenced how participants thought about physical activity. Or it is possible that participants had to walk despite physical limitations because it was the participants’ mode of transportation [47,48].

However, it is interesting to note that accelerometer-derived step counts tended to be higher than average among participants in this sample. In a 2022 meta-analysis of steps per day and all-cause mortality in U.S. adults, median steps per day among the highest quartile was 10,901 and associated with significantly lower risk of all-cause mortality than the lowest quartile [34]. We are uncertain how higher activity in the present study influences overall health or the exact reasons why activity is higher. Nevertheless, it may be a result of a variety of factors (e.g., transportation options).

The finding in the present study of low diet quality among adults living in transitional housing is not surprising, given that previous research in populations experiencing homelessness has shown them to have a low nutrient and food group intake [1,3,48]. Hoisington et al. [12] found a lower overall HEI-2020 score (64.0) among U.S. adult veterans experiencing homelessness compared to those not experiencing homelessness. The present study found an even lower overall HEI-2020 than the Hoisington et al. [12] study, suggesting even poorer diet quality among a general adult population experiencing homelessness and increased risk of negative health implications [49]. The less favorable physical measurements from the present study, combined with the low diet quality observed, are likely to put these individuals at even higher risk for chronic disease [50].

Potential reasons for the overall diet quality of participants in the present study being below the national norm may be related to the high rate of food insecurity observed and the barriers to healthy eating identified by participants. This study found that of the participants surveyed, about 70% were food insecure, which is not surprising based on previous research in this population [51,52]. With food insecurity, it is logical that diet quality would also be low, given questions within the USDA’s Six-Item Short Form ask about being able to consume balanced meals and having enough food [21]. Beyond this, participants in the present study provided qualitative data on barriers to healthy eating, with responses suggesting potential environmental reasons for this lower diet quality. In the transitional housing units where participants in the present study resided, there were 26 units for males and 12 units for females, with each resident area including one small, shared kitchen with one full-sized fridge and shared cabinets, thus likely contributing to participants’ perceptions of limited space to store food. Participants in the present study were also unable to utilize cooking tools such as a stove or knives and were limited to a microwave and basic utensils. Similar environmental constraints in a homeless shelter have previously been identified among transitional housing residents in Minneapolis, Minnesota [48], suggesting these barriers are common experiences among those experiencing homelessness in the U.S. Given previous research has found soup kitchens and shelters as a contributing factor to obesity among chronically homeless adults [2,4], this may also help to explain the high mean BMI observed in the present study.

Interestingly, the participants’ perceptions of general health in the present study were below the average for U.S. adults, but only by about four points [38]. This was unexpected considering the high rates of food insecurity, poorer body composition measures, elevated blood pressure levels, and low diet quality observed in the present study. One explanation for this may be that if participants previously lived in unhoused conditions (e.g., car, street, etc.) before transitioning into a temporary shelter at the local homeless resource center, accessing food in participants’ current living situation has improved, along with feelings of stability. In the present study, participants also had access to an on-site medical clinic, which may have contributed to general health scores not too far below US adults.

A recent meta-analysis study indicated that 67% of adults experiencing homelessness had been diagnosed with a mental health disorder, suggesting these disorders are commonly found in this population [53]. In the present study, participants’ responses indicated mean scores within the normal range for depression and stress, and in the mild range for anxiety. This lack of consistency with the meta-analysis may be explained by the small sample size in this pilot study, the self-reported nature of the instrument used in the present study rather than a medical diagnosis, and the meta-analysis including a more varied set of mental health disorders than the present study.

This study has several limitations. First, due to the nature of it being a pilot study, the sample size is small. This limited the authors’ analytical approach to descriptive statistics. This also prevented authors from stratifying absolute and relative grip strength by co-morbidities (e.g., BMI) and age, as was performed by Lawman et al. [31]. Future studies with larger sample sizes are needed to evaluate associations between variables. Second, it was only performed at one geographical location, with the sample identifying mostly as male and White. Thus, the results may not be similar to those experiencing homelessness at other locations across the U.S. or among more diverse audiences. In future studies, to collect data from a more diverse sample, researchers should recruit participants from multiple geographical areas and from a variety of community agencies providing services for unhoused and temporarily housed individuals. Third, participants were not asked about transportation access, so it is unknown whether their main modality was by foot, public transit, and/or private transportation, which would have offered insight into the high step count. Fourth, the term “physical activity” was not defined in the surveys. Thus, participants were left to interpret the term on their own, and the way they answered this question may have varied based on that interpretation. Lastly, the ASA24 was only collected on one day at baseline, which does not account for day-to-day variations observed in individuals’ diets and thereby limits its generalizability [10]. Future research should consider collecting multiple days of dietary recall to enhance the accuracy of diet quality measurements among transitional housing residents.

## 5. Conclusions

Individuals transitioning out of homelessness have less favorable health measures related to body composition, muscular strength, anxiety, and diet quality. Limited finances and cooking/storage facilities presented challenges for transitional housing residents to consume a healthy eating pattern. Although physical impediments were barriers identified by transitional housing residents in being physically active, accelerometer data suggested residents were highly active. Future interventions that are tailored to address the unique challenges faced by transitional housing residents are needed to promote optimal health among this population.

## Figures and Tables

**Table 1:** Baseline demographics of transitional housing residents participating in a wellness intervention program at a local homeless resource center (*n* = 29).

	Total, *n* (%)
Sex	
Male	17 (58.6)
Female	12 (41.4)
Age, years	
18–24	1 (3.4)
25–34	4 (13.8)
35–44	7 (24.1)
45–54	4 (13.8)
55–64	11 (37.9)
65+	2 (6.9)
Hispanic/Latino ^a^	
Yes	4 (14.8)
No	23 (85.2)
Race ^b^	
American Indian/Alaskan Native	1 (3.8)
Black/African American	1 (3.8)
White	23 (88.5)
Other ^c^	1 (3.8)
Food Security ^ad^	
High or Marginal	8 (29.6)
Low	13 (48.1)
Very Low	6 (22.2)

^a^ Missing data, *n* = 2. ^b^ Missing data, *n* = 3, and responses not selected: Asian, Native Hawaiian, or Pacific Islander. ^c^ Other responses included Mexican American (*n* = 1). ^d^ Data collected using the U.S. Household Food Security Survey Module: Six-Item Short Form [21].

**Table 2:** Baseline measurements of transitional housing residents participating in a wellness intervention program at a local homeless resource center (*n* = 29).

Physical Measurements	Mean	Std. Dev.	Reference Standards ^a^
Temperature (F)	97.7	1.0	97–99
Resting Heart Rate (bpm)	86.8	13.0	60–100
Oxygenation (%)	96.2	2.0	95–100
Blood Pressure			
Systolic (mmHg)	131.8	17.9	<120
Diastolic (mmHg)	85.2	13.3	<80
BMI (kg/m^2^)	31.8	8.6	18.5–24.9
Waist-to-Hip Ratio	0.9	0.1	
Male	1.0	0.1	<0.9 (M)
Female	0.9	0.1	<0.8 (F)
Body Fat (%)			
Male	25.8	6.1	19.8 ± 5.4 (M, all ages)
18–39 years	25.6	3.2	18.3 ± 4.8 (M, 18–39 years)
40–59 years	26.1	9.2	20.5± 5.3 (M, 40–59 years)
60+ years	25.4	3.2	24.0 ± 5.3 (M, 60+ years)
Female	40.5	9.4	28.7 ± 6.4 (F, all ages)
18–39 years	36.6	10.9	26.5 ± 5.1 (F, 18–39 years)
40–59 years	44.4	6.0	28.3 ± 5.8 (F, 40–59 years)
60+ years	--	--	35.2 ± 6.3(F, 60+ years)
Body Fat Mass Index (kg/m^2^)			
Male	7.7	3.1	4.9 ± 1.8 (M, all ages)
18–39 years	7.7	1.5	4.3 ± 1.5 (M, 18–39 years)
40–59 years	8.3	4.6	5.1 ± 1.7 (M, 40–59 years)
60+ years	6.9	1.6	6.2 ± 1.9 (M, 60+ years)
Female	15.3	7.7	6.6 ± 2.4 (F, all ages)
18–39 years	13.8	9.2	5.7 ± 1.7 (F, 18–39 years)
40–59 years	16.8	6.4	6.6 ± 2.2 (F, 40–59 years)
60+ years	--	--	9.1 ± 2.9 (F, 60+ years)
Fat-free Mass Index (kg/m^2^)			
Male	21.2	2.3	19.1 ± 1.4 (M, all ages)
18–39 years	22.1	1.1	19.0 ± 1.3 (M, 18–39 years)
40–59 years	21.3	3.0	19.4 ± 1.4 (M, 40–59 years)
60+ years	19.9	2.1	19.1 ± 1.6 (M, 60+ years)
Female	20.4	3.7	15.9 ± 1.3 (F, all ages)
18–39 years	20.8	4.3	15.6 ± 1.1 (F, 18–39 years)
40–59 years	20.0	3.5	16.2 ± 1.3 (F, 40–59 years)
60+ years	--	--	16.2 ± 1.7 (F, 60+ years)
Absolute Grip Strength (kg) ^b^			
Male	77.1	19.4	89.7 ± 0.8 (M)
Female	53.0	15.7	56.1 ± 0.5 (F)
Relative Grip Strength (kg/BMI) ^b^			
Male	2.7	0.8	3.2 ± 0.5 (M)
Female	1.6	0.6	2.0 ± 0.02 (F)
Physical Activity ^c^			
Steps counts (per day)	14,334.0	14,297.8	6000–10,000
Steps counts (per day) *	11,524.3	3951.6	6000–10,000

F = Fahrenheit; bpm = beats per minute; BMI = Body Mass Index; F = female; M = male; ^a^ Reference standards for the following measures are: body temperature [22]; resting heart rate [23]; blood oxygenation [24]; systolic and diastolic blood pressure [26]; BMI [27]; sex-specific waist-to-hip ratio [28]; sex- and age- specific body fat percentage [29]; sex- and age-specific body fat index and fat-free mass index [29]; sex-specific absolute and relative grip strength [31]; and daily step count [34]; ^b^ missing data, *n* = 1 (male); ^c^ missing data, *n* = 5; * with *n* = 1 outlier removed.

**Table 3:** Healthy Eating Index-2020 components of transitional housing residents participating in a wellness intervention program at a local homeless resource center (*n* = 29).

HEI-2020 Components	Maximum HEI Score	Mean Score	Std. Dev.
Adequacy			
Total Fruits	5	1.4	1.9
Whole Fruits	5	1.3	2.1
Total Vegetables	5	2.4	2.0
Greens and Beans	5	1.9	2.2
Whole Grains	10	0.8	1.7
Dairy	10	6.3	3.5
Total Protein Foods	5	4.2	1.5
Seafood and Plant Proteins	5	1.5	2.1
Fatty Acids	10	1.2	2.6
Moderation			
Refined Grains	10	6.2	3.6
Sodium	10	3.9	3.7
Added Sugars	10	7.2	3.2
Fatty Acids	10	1.3	2.8
Total HEI Score	100	39.7	10.0

HEI = Healthy Eating Index.

**Table 4:** Perceptions of health-related behaviors among transitional housing residents participating in a wellness intervention program at a local homeless resource center (*n* = 29).

	Mean	Std. Dev.	Reference Standards/ Classifications
Health-Related Quality of Life (SF-36) ^a,b^			
Physical functioning	75.9	26.8	70.6 ± 27.4
Role limitations due to physical health	57.4	37.9	53.0 ± 40.8
Role limitations due to emotional problems	55.6	43.4	65.8 ± 40.7
Energy/fatigue	51.7	21.2	52.2 ± 22.4
Emotional well-being	61.9	18.7	70.4 ± 22.0
Social functioning	61.6	27.7	78.8 ± 25.4
Pain	55.3	25.7	70.8 ± 25.5
General health	52.9	29.8	57.0 ± 21.1
DASS-42 ^c,d^			
Depression	8.6	8.7	Normal = 0–9 Mild = 10–13 Moderate = 14–20 Severe = 21–27 Extremely severe = 28+
Anxiety	9.3	9.3	Normal = 0–7 Mild = 8–9 Moderate = 10–14 Severe = 15–19 Extremely severe = 20+
Stress	10.9	9.5	Normal = 0–14 Mild = 15–18 Moderate = 19–25 Severe = 26–33 Extremely severe = 34+
Create Better Health Items ^d^			
I stretch my food dollars so there is food to last the entire month. ^a^	3.2	1.5	--
I choose a variety of foods based on MyPlate recommendations. ^a^	2.3	1.5	--
I use the nutrition facts label to make food choices. ^a^	2.6	1.3	--
I shop with a grocery list. ^a^	3.0	1.5	--
I follow USDA food safety recommendations. ^e^	2.5	1.5	--
I adjust meals to use foods I already have at home. ^e^	3.7	1.1	--

DASS = Depression Anxiety Stress Scale; USDA = United States Department of Agriculture. ^a^ Missing data, *n* = 2; ^b^ reference standards from RAND [38]; ^c^ scores are summed across items measuring depression, anxiety, and stress [39]; ^d^ missing data, *n* = 3; ^e^ responses based on a 5-point Likert scale: 1 = never, 2 = seldom, 3 = sometimes, 4 = usually, and 5 = always.

## Data Availability

The raw data supporting the conclusions of this article will be made available by the authors on request.

## Abbreviations

The following abbreviations are used in this manuscript:

U.S.	United States
BMI	Body Mass Index
SNAP-Ed	Supplementation Nutrition Assistance Program Education
USDA	United States Department of Agriculture
ASA24	Automated Self-Administered Dietary Assessment Tool
HRQOL	Health-Related Quality of Life
DASS	Depression, Anxiety, Stress Scale
SPSS	Statistical Packages for Social Scientists

## References

[1] Derrickson J., Gans D.A. (1996). Assessment of Dietary Intake and Food-Related Behaviors of Gatekeepers in Homeless Families in Hawaii. J. Nutr. Education.

[2] Richards R., Smith C. (2010). Investigation of the Hunger–Obesity Paradigm Among Shelter-Based Homeless Women Living in Minnesota. J. Hunger. Environ. Nutr..

[3] Lyles C.R., Drago-Ferguson S., Lopez A., Seligman H.K. (2013). Nutritional Assessment of Free Meal Programs in San Francisco. Prev. Chronic Dis..

[4] Martins D.C., Gorman K.S., Miller R.J., Murphy L., Sor S., Martins J.C., Vecchiarelli M.L. (2015). Assessment of Food Intake, Obesity, and Health Risk among the Homeless in Rhode Island. Public Health Nurs..

[5] Johnson L.J., Myung E., McCool A.C., Champaner E.I. (2009). Nutrition Education for Homeless Women—Challenges and Opportunities: A Pilot Study. J. Foodserv. Bus. Res..

[6] Kiernan S., Ní Cheallaigh C., Murphy N., Dowds J., Broderick J. (2021). Markedly Poor Physical Functioning Status of People Experiencing Homelessness Admitted to an Acute Hospital Setting. Sci. Rep..

[7] Baggett T.P., Liauw S.S., Hwang S.W. (2018). Cardiovascular Disease and Homelessness. J. Am. Coll. Cardiol..

[8] Tsai J., Rosenheck R.A. (2013). Obesity Among Chronically Homeless Adults: Is It a Problem?. Public Health Rep..

[9] Koh K.A., Hoy J.S., O’Connell J.J., Montgomery P. (2012). The Hunger–Obesity Paradox: Obesity in the Homeless. J. Urban Health.

[10] Seale J.V., Fallaize R., Lovegrove J.A. (2016). Nutrition and the Homeless: The Underestimated Challenge. Nutr. Res. Rev..

[11] U.S. Department of Agriculture and U.S. Department of Health and Human Services (2020). The Dietary Guidelines for Americans, 2020–2025.

[12] Hoisington A.J., Stearns-Yoder K.A., Stamper C.E., Holliday R., Brostow D.P., Penzenik M.E., Forster J.E., Postolache T.T., Lowry C.A., Brenner L.A. (2024). Association of Homelessness and Diet on the Gut Microbiome: A United States-Veteran Microbiome Project (US-VMP) Study. mSystems.

[13] Cornier M.-A., Després J.-P., Davis N., Grossniklaus D.A., Klein S., Lamarche B., Lopez-Jimenez F., Rao G., St-Onge M.-P., Towfighi A. (2011). Assessing Adiposity: A Scientific Statement From the American Heart Association. Circulation.

[14] Khan I., Chong M., Le A., Mohammadi-Shemirani P., Morton R., Brinza C., Kiflen M., Narula S., Akhabir L., Mao S. (2023). Surrogate Adiposity Markers and Mortality. JAMA Netw. Open.

[15] Ruiz Coronel A., Fossión R., Sauri García J. (2019). Physiological Frailty in Chronically Homeless Young Adults Determined by Handgrip Strength. Soc. Med..

[16] Vaishya R., Misra A., Vaish A., Ursino N., D’Ambrosi R. (2024). Hand Grip Strength as a Proposed New Vital Sign of Health: A Narrative Review of Evidences. J. Health Popul. Nutr..

[17] Dawes J., Rogans-Watson R., Broderick J. (2024). ‘You Can Change Your Life through Sports’—Physical Activity Interventions to Improve the Health and Well-Being of Adults Experiencing Homelessness: A Mixed-Methods Systematic Review. Br. J. Sports Med..

[18] Kendzor D.E., Allicock M., Businelle M.S., Sandon L.F., Gabriel K.P., Frank S.G. (2017). Evaluation of a Shelter-Based Diet and Physical Activity Intervention for Homeless Adults. J. Phys. Act. Health.

[19] Strongo K., Coombs C., LeCheminant J.D., Merrill C., Jenkins C., LeBlanc H., Smith M.W., Bell M., Skidmore B.K., Richards R. (2025). Formative Qualitative Research Informs Tailoring SNAP-Ed Curriculum for Transitional Housing Residents. J. Nutr. Educ. Behav..

[20] Ajeen R., Ajeen D., Wisdom J.P., Greene J.A., Lepage T., Sjoelin C., Melvin T., Hagan T.E., Hunter K.F., Peters A. (2022). The Impact of Trauma-Informed Design on Psychological Well-Being in Homeless Shelters. Psychol. Serv..

[21] USDA ERS—Survey Tools. https://www.ers.usda.gov/topics/food-nutrition-assistance/food-security-in-the-u-s/survey-tools/#adult.

[22] Medline Plus Body Temperature Norms. https://medlineplus.gov/ency/article/001982.htm.

[23] Mayo Clinic What’s a Normal Resting Heart Rate?. https://www.mayoclinic.org/healthy-lifestyle/fitness/expert-answers/heart-rate/faq-20057979.

[24] Cleveland Clinic Blood Oxygen Level. https://my.clevelandclinic.org/health/diagnostics/22447-blood-oxygen-level.

[25] Campbell N.R.C., Paccot Burnens M., Whelton P.K., Angell S.Y., Jaffe M.G., Cohn J., Espinosa Brito A., Irazola V., Brettler J.W., Roccella E.J. (2022). 2021 World Health Organization Guideline on Pharmacological Treatment of Hypertension: Policy Implications for the Region of the Americas. Lancet Reg. Health.

[26] American Heart Association Understanding Blood Pressure Readings. https://www.heart.org/en/health-topics/high-blood-pressure/understanding-blood-pressure-readings.

[27] Centers for Disease Control and Prevention Adult BMI Categories. https://www.cdc.gov/bmi/adult-calculator/bmi-categories.html.

[28] World Health Organization (2011). Waist Circumference and Waist-to-Hip Ratio: Report of a WHO Expert Consultation. https://www.who.int/publications/i/item/9789241501491.

[29] Kyle U.G., Schutz Y., Dupertuis Y.M., Pichard C. (2003). Body Composition Interpretation. Nutrition.

[30] Norman K., Stobäus N., Gonzalez M.C., Schulzke J.-D., Pirlich M. (2011). Hand Grip Strength: Outcome Predictor and Marker of Nutritional Status. Clin. Nutr..

[31] Lawman H.G., Troiano R.P., Perna F.M., Wang C.-Y., Fryar C.D., Ogden C.L. (2016). Associations of Relative Handgrip Strength and Cardiovascular Disease Biomarkers in U.S. Adults, 2011–2012. Am. J. Prev. Med..

[32] Neishabouri A., Nguyen J., Samuelsson J., Guthrie T., Biggs M., Wyatt J., Cross D., Karas M., Migueles J.H., Khan S. (2022). Quantification of Acceleration as Activity Counts in ActiGraph Wearable. Sci. Rep..

[33] Gao Z., Liu W., McDonough D.J., Zeng N., Lee J.E. (2021). The Dilemma of Analyzing Physical Activity and Sedentary Behavior with Wrist Accelerometer Data: Challenges and Opportunities. J. Clin. Med..

[34] Paluch A.E., Bajpai S., Bassett D.R., Carnethon M.R., Ekelund U., Evenson K.R., Galuska D.A., Jefferis B.J., Kraus W.E., Lee I.-M. (2022). Daily Steps and All-Cause Mortality: A Meta-Analysis of 15 International Cohorts. Lancet Public Health.

[35] National Cancer Institute Automated Self-Administered 24-Hour (ASA24®) Dietary Assessment Tool. https://epi.grants.cancer.gov/asa24/.

[36] National Cancer Institute Overview & Background of the Healthy Eating Index. https://epi.grants.cancer.gov/hei/.

[37] U.S. Department of Agriculture Healthy Eating Index Scores for Americans. https://www.fns.usda.gov/cnpp/hei-scores-americans.

[38] RAND 36-Item Short Form Survey (SF-36) Scoring Instructions. https://www.rand.org/health-care/surveys_tools/mos/36-item-short-form/scoring.html.

[39] Psychology Foundation of Australia Depression Anxiety Stress Scales (DASS). https://www2.psy.unsw.edu.au/dass/.

[40] National Cancer Institute Healthy Eating Index SAS Code. https://epi.grants.cancer.gov/hei/sas-code.html.

[41] Troiano R.P., Berrigan D., Dodd K.W., Mâsse L.C., Tilert T., Mcdowell M. (2008). Physical Activity in the United States Measured by Accelerometer. Med. Sci. Sports Exerc..

[42] Hsieh H.-F., Shannon S.E. (2005). Three Approaches to Qualitative Content Analysis. Qual. Health Res..

[43] Abernathy R., Black D. (1996). Healthy Body Weights: An Alternative Perspective. Am. J. Clin. Nutr..

[44] Koning M., Vink J.M., Renders C., Notten N., Eisinga R., Larsen J.K. (2021). Is the Prospective Link between Parental Stress and Adolescent Snack Intake or Weight Outcome Mediated by Food Parenting Practices?. Nutrients.

[45] Centers for Disease Control and Prevention Consequences of Obesity. https://www.cdc.gov/obesity/basics/consequences.html.

[46] Cohen C.I., Benyaminov R., Rahman M., Ngu D., Reinhardt M. (2023). Frailty. Med. Clin. North Am..

[47] Brallier S., Southworth S., Ryan B. (2019). Rolling Forward: Addressing Needs in the Homeless Community. J. Soc. Distress Homelessness.

[48] Richards R., Smith C. (2006). Shelter Environment and Placement in Community Affects Lifestyle Factors among Homeless Families in Minnesota. Am. J. Health Promot..

[49] Morze J., Danielewicz A., Hoffmann G., Schwingshackl L. (2020). Diet Quality as Assessed by the Healthy Eating Index, Alternate Healthy Eating Index, Dietary Approaches to Stop Hypertension Score, and Health Outcomes: A Second Update of a Systematic Review and Meta-Analysis of Cohort Studies. J. Acad. Nutr. Diet..

[50] Hacker K. (2024). The Burden of Chronic Disease. Mayo Clin. Proc. Innov. Qual. Outcomes.

[51] Fitzpatrick K.M., Willis D.E. (2021). Homeless and Hungry: Food Insecurity in the Land of Plenty. Food Secur..

[52] Hernandez D.C., Daundasekara S.S., Arlinghaus K.R., Tobar N., Reitzel L.R., Kendzor D.E., Businelle M.S. (2019). Cumulative Risk Factors Associated with Food Insecurity among Adults Who Experience Homelessness. Health Behav. Res..

[53] Barry R., Anderson J., Tran L., Bahji A., Dimitropoulos G., Ghosh S.M., Kirkham J., Messier G., Patten S.B., Rittenbach K. (2024). Prevalence of Mental Health Disorders Among Individuals Experiencing Homelessness: A Systematic Review and Meta-Analysis. JAMA Psychiatry.

